# Supplementary material for: Practical Determination of the Solubility Parameters of 1-Alkyl-3-methylimidazolium Bromide ([CnC1im]Br, n = 5, 6, 7, 8) Ionic Liquids by Inverse Gas Chromatography and the Hansen Solubility Parameter
Source: Molecules. 2019 Apr 5;24(7):1346. doi: 10.3390/molecules24071346 (PMC6479879; doi:10.3390/molecules24071346)
Supplement: Supplementary file 1 [file molecules-24-01346-s001.pdf]

Supplementary Materials

Table S1. The specific retention volume, V_g^0, between the probe and IL at various temperatures for the hypothetical liquids at zero pressure.

Probes	ILs	V_g^0				
		**303.15 K**	**313.15 K**	**323.15 K**	**333.15 K**	**343.15 K**
n-C_6	[C_5C_1im]Br	7.74	5.73	4.17	3.58	2.73
	[C_6C_1im]Br	9.20	6.32	4.45	3.96	3.15
	[C_7C_1im]Br	11.5	8.83	6.21	4.48	3.52
	[C_8C_1im]Br	22.7	15.5	10.9	7.86	6.35
n-C_7	[C_5C_1im]Br	23.6	16.0	10.9	7.79	5.78
	[C_6C_1im]Br	26.4	18.0	12.7	8.83	6.21
	[C_7C_1im]Br	34.4	24.6	16.5	11.8	8.57
	[C_8C_1im]Br	61.8	40.8	27.4	19.1	13.8
n-C_8	[C_5C_1im]Br	67.0	42.5	27.4	18.2	12.5
	[C_6C_1im]Br	79.8	52.2	34.3	22.5	15.1
	[C_7C_1im]Br	94.8	70.8	45.0	29.0	19.6
	[C_8C_1im]Br	176	106	67.7	44.5	30.2
n-C_9	[C_5C_1im]Br	171	103	64.1	41.1	27.2
	[C_6C_1im]Br	221	136	85.4	54.7	36.7
	[C_7C_1im]Br	261	158	99.2	64.0	42.5
	[C_8C_1im]Br	422	250	154	99.5	66.3
n-C_{10}	[C_5C_1im]Br	458	258	154	94.4	60.1
	[C_6C_1im]Br	582	336	200	122	79.0
	[C_7C_1im]Br	675	390	228	140	89.6
	[C_8C_1im]Br	1115	618	365	224	142
n-C_{11}	[C_5C_1im]Br	1166	619	347	202	125
	[C_6C_1im]Br	1451	783	435	257	159
	[C_7C_1im]Br	1673	906	506	295	182
	[C_8C_1im]Br	2819	1481	828	481	294
n-C_{12}	[C_5C_1im]Br	2992	1512	776	438	258
	[C_6C_1im]Br	3693	1837	966	536	317
	[C_7C_1im]Br	4113	2094	1096	628	361
	[C_8C_1im]Br	6924	3469	1843	1032	582
benzene	[C_5C_1im]Br	280	187	131	93.3	69.2
	[C_6C_1im]Br	271	183	125	89.6	66.6
	[C_7C_1im]Br	264	177	122	85.4	64.5
	[C_8C_1im]Br	259	171	117	83.6	62.4
toluene	[C_5C_1im]Br	558	357	233	166	117
	[C_6C_1im]Br	532	344	227	165	114
	[C_7C_1im]Br	504	331	241	161	111
	[C_8C_1im]Br	563	360	233	157	109
o-xylene	[C_5C_1im]Br	1697	1022	619	462	301
	[C_6C_1im]Br	1648	1016	615	439	293
	[C_7C_1im]Br	1633	1000	587	437	284
	[C_8C_1im]Br	1568	981	539	437	279

m-xylene	[C_5C_1im]Br	1045	637	394	303	199
	[C_6C_1im]Br	1066	627	400	290	192
	[C_7C_1im]Br	1303	790	481	304	200
	[C_8C_1im]Br	1263	764	475	306	205
p-xylene	[C_5C_1im]Br	1073	646	402	304	200
	[C_6C_1im]Br	1079	673	431	292	200
	[C_7C_1im]Br	1283	769	469	298	197
	[C_8C_1im]Br	1231	745	463	299	201
ethyl benzene	[C_5C_1im]Br	999	604	388	291	193
	[C_6C_1im]Br	1009	624	396	279	188
	[C_7C_1im]Br	1166	734	449	286	191
	[C_8C_1im]Br	1139	704	441	286	193
n-propyl benzene	[C_5C_1im]Br	1736	1034	620	454	293
	[C_6C_1im]Br	2411	1392	832	513	334
	[C_7C_1im]Br	2292	1327	801	501	322
	[C_8C_1im]Br	2409	1402	840	524	340
n-butyl benzene	[C_5C_1im]Br	3613	2002	1160	815	506
	[C_6C_1im]Br	5364	2949	1652	972	606
	[C_7C_1im]Br	5410	2969	1741	1006	617
	[C_8C_1im]Br	5820	3210	1823	1089	677
cyclohexene	[C_5C_1im]Br	48.3	38.2	26.4	18.7	14.7
	[C_6C_1im]Br	60.6	42.5	30.1	21.8	16.0
	[C_7C_1im]Br	57.8	42.7	30.0	21.9	15.9
	[C_8C_1im]Br	63.5	45.7	32.3	23.5	17.6
octene	[C_5C_1im]Br	66.3	46.4	29.3	19.5	13.9
	[C_6C_1im]Br	88.1	56.8	37.1	24.9	17.4
	[C_7C_1im]Br	105	70.0	44.6	30.4	20.7
	[C_8C_1im]Br	137	88.1	56.0	37.4	26.0
pentanone	[C_5C_1im]Br	569	378	245	164	112
	[C_6C_1im]Br	503	326	213	145	102
	[C_7C_1im]Br	451	301	196	130	89.4
	[C_8C_1im]Br	423	274	178	121	84.5
3-pentanone	[C_5C_1im]Br	565	361	232	156	109
	[C_6C_1im]Br	497	321	208	143	100
	[C_7C_1im]Br	446	294	192	128	87.8
	[C_8C_1im]Br	414	266	174	118	83.3
thiophene	[C_5C_1im]Br	828	527	351	231	160
	[C_6C_1im]Br	713	455	301	205	143
	[C_7C_1im]Br	557	355	237	159	111
	[C_8C_1im]Br	471	298	189	124	81.4
nitromethane	[C_5C_1im]Br	3217	2023	1278	849	574
	[C_6C_1im]Br	2469	1561	1003	678	458
	[C_7C_1im]Br	1742	1117	723	474	325
	[C_8C_1im]Br	1305	866	576	390	271
methanol	[C_5C_1im]Br	3137	1981	1273	844	589
	[C_6C_1im]Br	2668	1649	1082	732	530
	[C_7C_1im]Br	1835	1159	746	495	337

	$[C_8C_1im]Br$	1463	960	627	416	288
ethanol	$[C_5C_1im]Br$	3670	2207	1375	902	617
	$[C_6C_1im]Br$	3205	1942	1205	762	497
	$[C_7C_1im]Br$	2426	1443	908	576	380
	$[C_8C_1im]Br$	1994	1267	797	512	343
propanol	$[C_5C_1im]Br$	7997	4693	2727	1670	1067
	$[C_6C_1im]Br$	7343	4241	2542	1537	960
	$[C_7C_1im]Br$	5991	3359	1970	1194	760
	$[C_8C_1im]Br$	4872	2965	1792	1093	707
isopropanol	$[C_5C_1im]Br$	3046	1817	1093	684	455
	$[C_6C_1im]Br$	2828	1670	1021	643	418
	$[C_7C_1im]Br$	2270	1342	809	503	324
	$[C_8C_1im]Br$	1930	1189	728	459	300
butanol	$[C_5C_1im]Br$	18057	10073	5594	3264	2013
	$[C_6C_1im]Br$	16635	8962	4869	2810	1724
	$[C_7C_1im]Br$	15202	8031	4473	2586	1614
	$[C_8C_1im]Br$	13002	7410	4162	2421	1492
2-butanol	$[C_5C_1im]Br$	6260	3394	1982	1211	766
	$[C_6C_1im]Br$	5638	3066	1765	1089	704
	$[C_7C_1im]Br$	5059	2824	1617	968	603
	$[C_8C_1im]Br$	4325	2562	1503	913	570
isobutanol	$[C_5C_1im]Br$	12090	6409	3735	2247	1375
	$[C_6C_1im]Br$	10754	5903	3313	1941	1191
	$[C_7C_1im]Br$	9465	5187	2957	1734	1064
	$[C_8C_1im]Br$	8099	4729	2735	1630	1005

Standard uncertainties are as follow: u (T) = ±0.5 K, u (V_g^0) = 0.03.

Table S2. Probe description table.

Chemical name	CASRN	source	initial mole fraction purity	Water content	Final mole fraction purity
$[C_5C_1im]Br$	343851-31-0	Chengjie Chemical Co.Ltd.	0.98	540 ppm	0.999
$[C_6C_1im]Br$	85100-78-3	Chengjie Chemical Co.Ltd.	0.98	360 ppm	0.999
$[C_7C_1im]Br$	343851-32-1	Chengjie Chemical Co.Ltd.	0.98	480 ppm	0.999
$[C_8C_1im]Br$	61545-99-1	Chengjie Chemical Co.Ltd.	0.98	570 ppm	0.999
n-C_9	111-84-2	J&K Scientific Ltd.	0.99		
n-C_{10}	124-18-5	J&K Scientific Ltd.	0.98		
n-C_{11}	1120-21-4	J&K Scientific Ltd.	0.98		
n-C_{12}	112-40-3	J&K Scientific Ltd.	0.98		
cyclopentane	287-92-3	J&K Scientific Ltd.	0.99		
cyclohexane	110-82-7	J&K Scientific Ltd.	0.995		
2,2,4-trimethylpentane	540-84-1	J&K Scientific Ltd.	0.99		
benzene	71-43-2	J&K Scientific Ltd.	0.995		
toluene	108-88-3	J&K Scientific Ltd.	0.995		
o-xylene	95-47-6	J&K Scientific Ltd.	0.98		
m-xylene	108-38-3	J&K Scientific Ltd.	0.99		
p-xylene	106-42-3	J&K Scientific Ltd.	0.98		
ethyl benzene	64-04-0	J&K Scientific Ltd.	0.99		
n-propyl benzene	103-65-1	J&K Scientific Ltd.	0.98		

n-butyl benzene	104-51-8	J&K Scientific Ltd.	0.99
acetone	67-64-1	J&K Scientific Ltd.	0.995
nitromethane	75-52-5	J&K Scientific Ltd.	0.995
cyclohexene	110-83-8	J&K Scientific Ltd.	0.99
octene	111-66-0	J&K Scientific Ltd.	0.99
methyl ethyl ketone	78-93-3	J&K Scientific Ltd.	0.99
cyclohexanone	108-94-1	J&K Scientific Ltd.	0.99
dichloromethane	75-09-2	J&K Scientific Ltd.	0.995
chloroform	67-66-3	J&K Scientific Ltd.	0.995
carbon tetrachloride	56-23-5	J&K Scientific Ltd.	0.995
tetrahydrofuran	109-99-9	J&K Scientific Ltd.	0.99
pyridine	110-86-1	J&K Scientific Ltd.	0.99
thiophene	110-02-1	J&K Scientific Ltd.	0.99
propylene oxide	75-56-9	J&K Scientific Ltd.	0.99
1,4-dioxane	123-91-1	J&K Scientific Ltd.	0.99
pentanone	107-87-9	J&K Scientific Ltd.	0.98
3-pentanone	96-22-0	J&K Scientific Ltd.	0.99
methanol	67-56-1	J&K Scientific Ltd.	0.999
ethanol	64-17-5	J&K Scientific Ltd.	0.997

propanol	71-23-8	J&K Scientific Ltd.	0.995
isopropanol	67-63-0	J&K Scientific Ltd.	0.99
butanol	71-36-3	J&K Scientific Ltd.	0.99
2-butanol	78-92-2	J&K Scientific Ltd.	0.995
isobutanol	78-83-1	J&K Scientific Ltd.	0.99
acetonitrile	75-05-8	J&K Scientific Ltd.	0.99
methyl propionate	554-12-1	J&K Scientific Ltd.	0.99
ethyl propionate	105-37-3	J&K Scientific Ltd.	0.99
methyl formate	107-31-3	J&K Scientific Ltd.	0.99
methyl acetate	79-20-9	J&K Scientific Ltd.	0.98
ethyl acetate	141-78-6	J&K Scientific Ltd.	0.995
n-C_6	110-54-3	J&K Scientific Ltd.	0.998
n-C_7	142-82-5	J&K Scientific Ltd.	0.985
n-C_8	111-65-9	J&K Scientific Ltd.	0.98
n-C_5	109-66-0	J&K Scientific Ltd.	0.995
ethylene glycol	107-21-1	J&K Scientific Ltd.	0.99
N, N-dimethylformamide	68-12-2	J&K Scientific Ltd.	0.99
dimethyl sulfoxide	2206-27-1	J&K Scientific Ltd.	0.99

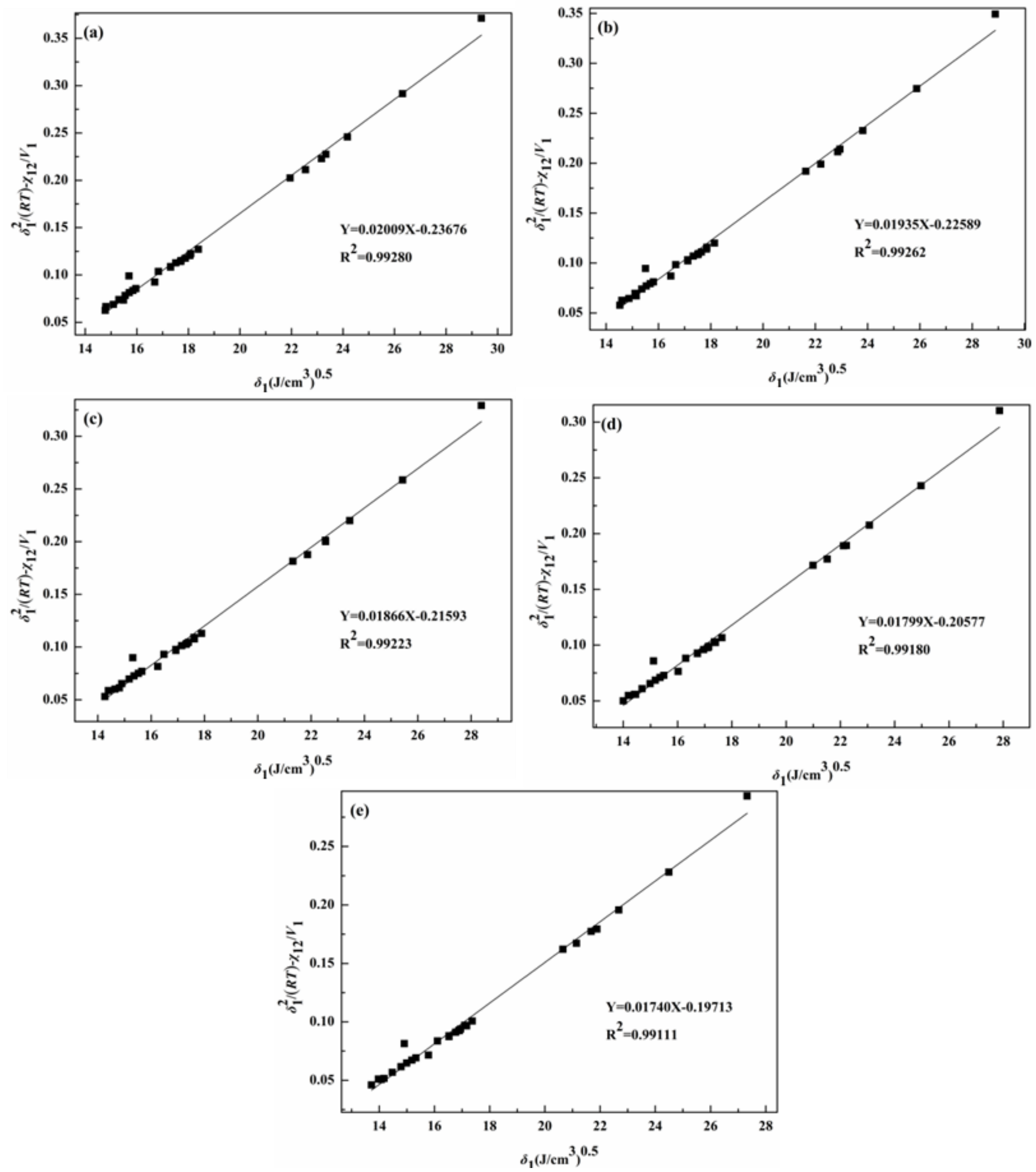

Figure S1 Variation of the term $\delta_1^2/RT - \chi_{12}/V_1$ with the solubility parameters of the solvent δ_1 in $[C_6C_1im]Br$ (a) at 303.15 K; (b) at 313.15 K; (c) at 323.15 K; (d) at 333.15 K.; (e) at 343.15 K.

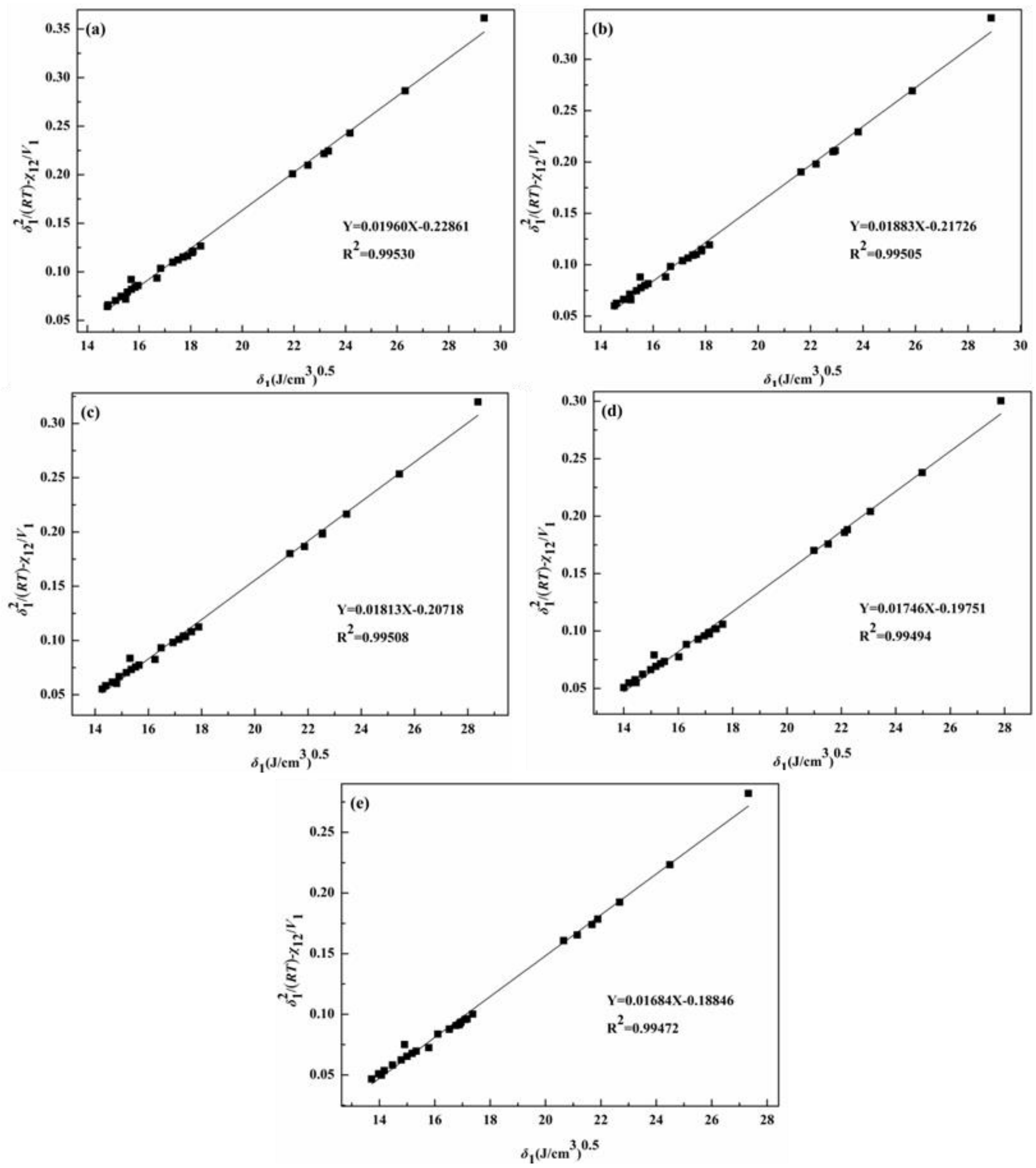

Figure S2 Variation of the term $\delta_1^2/RT - \chi_{12}/V_1$ with the solubility parameters of the solvent δ_1 in $[C_7C_1im]Br$ (a) at 303.15 K; (b) at 313.15 K; (c) at 323.15 K; (d) at 333.15 K.; (e) at 343.15 K.

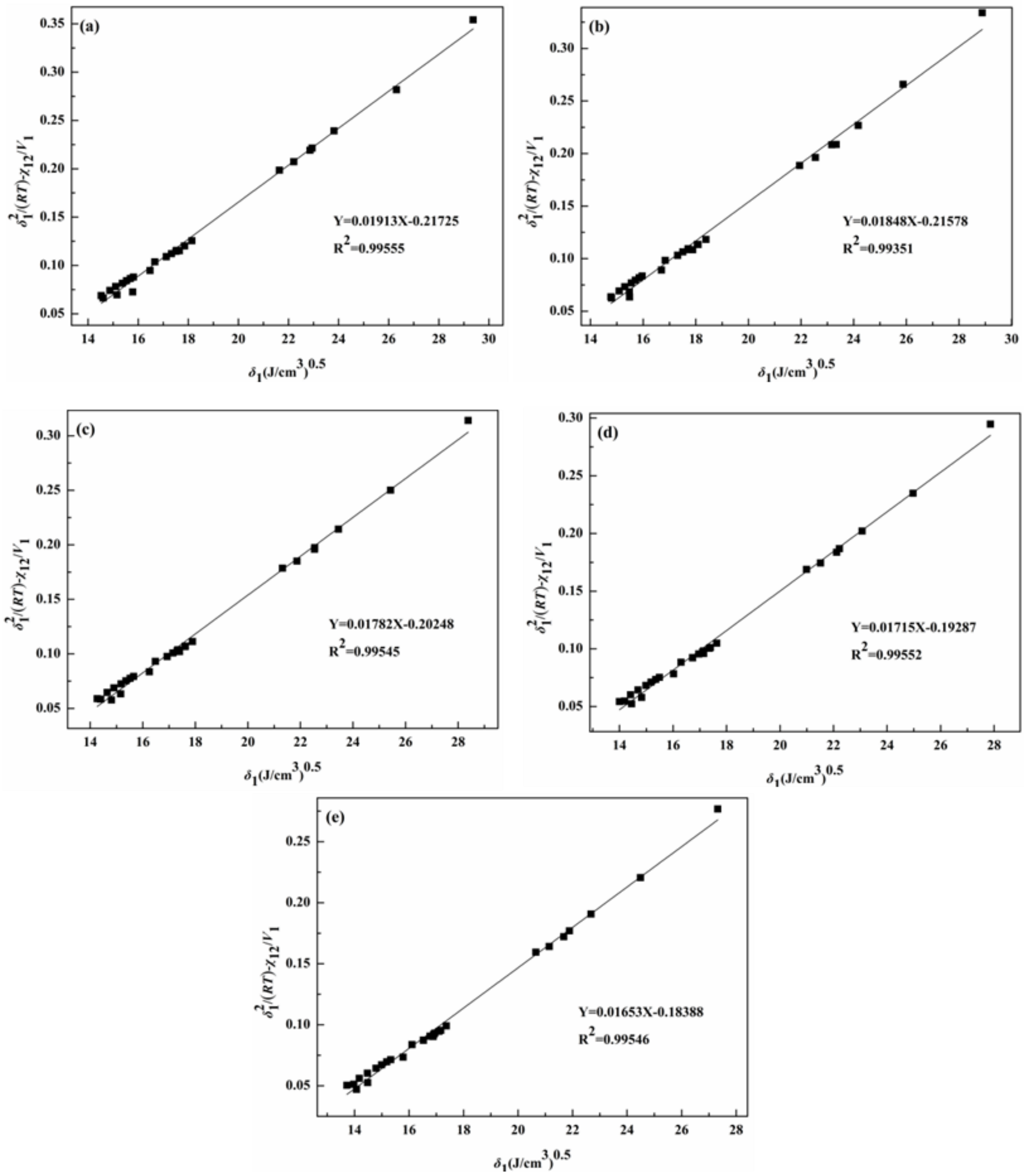

Figure S3 Variation of the term $\delta_1^2/RT - \chi_{12}/V_1$ with the solubility parameters of the solvent δ_1 in $[C_8C_1im]Br$ (a) at 303.15 K; (b) at 313.15 K; (c) at 323.15 K; (d) at 333.15 K.; (e) at 343.15 K.

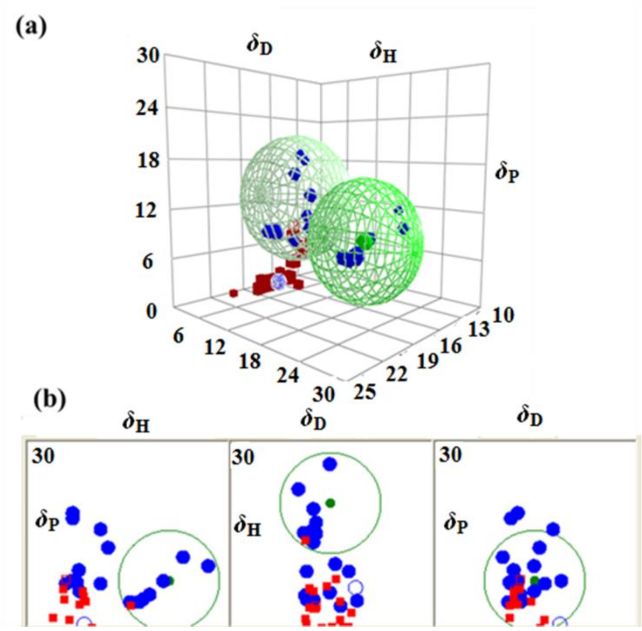

Figure S4 (a) The 3D graph with the coordinates of [C_6C_1im]Br; (b) The 2D graphs corresponding to the 3D graph of [C_6C_1im]Br.

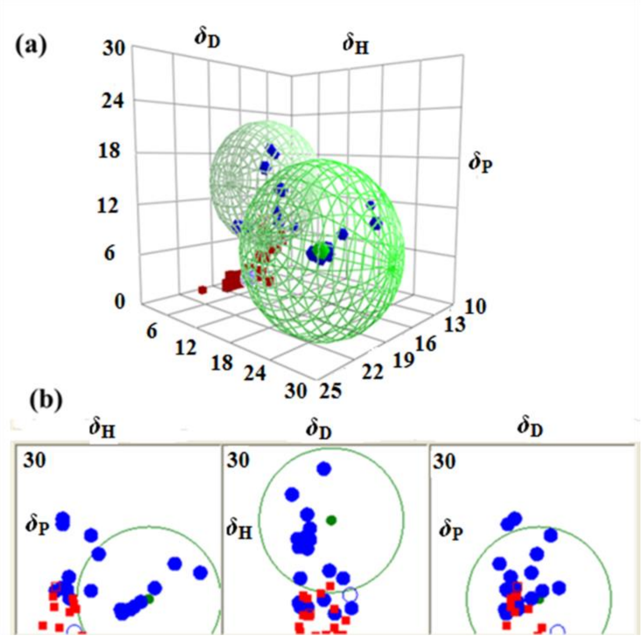

Figure S5 (a)The 3D graph with the coordinates of $[C_7C_1im]Br$; (b) The 2D graphs corresponding to the 3D graph of $[C_7C_1im]Br$.

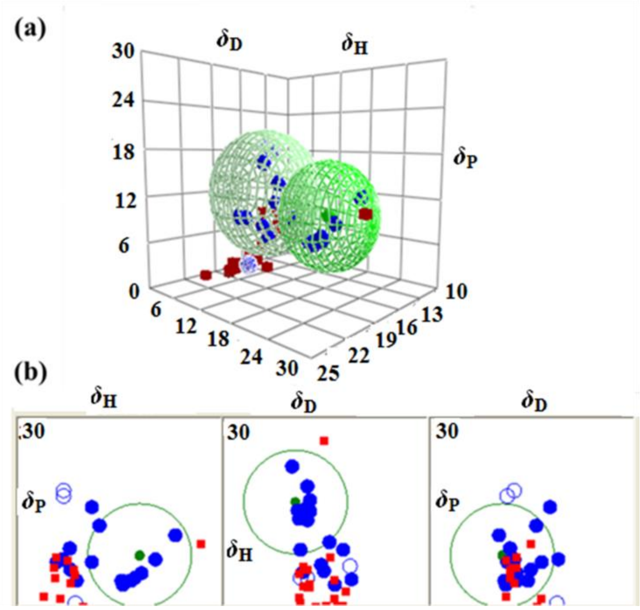

Figure S6 (a) The 3D graph with the coordinates of [C_8C_1im]Br; (b) The 2D graphs corresponding to the 3D graph of [C_8C_1im]Br.
